# Rubber Material Property Prediction Using Electron Microscope Images of Internal Structures Taken under Multiple Conditions

**DOI:** 10.3390/s21062088

**Published:** 2021-03-16

**Authors:** Ren Togo, Naoki Saito, Keisuke Maeda, Takahiro Ogawa, Miki Haseyama

**Affiliations:** 1 Education and Research Center for Mathematical and Data Science, Hokkaido University, N-12, W-7, Kita-ku, Sapporo 060-0812, Japan; 2 Department of Creative Engineering, National Institute of Technology, Kushiro College, Otanoshike-Nishi 2-32-1, Kushiro 084-0916, Japan; n-saito@kushiro-ct.ac.jp; 3 Office of Institutional Research, Hokkaido University, N-8, W-5, Kita-ku, Sapporo 060-0808, Japan; maeda@lmd.ist.hokudai.ac.jp; 4 Faculty of Information Science and Technology, Hokkaido University, N-14, W-9, Kita-ku, Sapporo 060-0814, Japan; ogawa@lmd.ist.hokudai.ac.jp (T.O.); miki@ist.hokudai.ac.jp (M.H.)

**Keywords:** rubber materials, property prediction, electron microscope images, Dempster–Shafer evidence theory

## Abstract

A method for prediction of properties of rubber materials utilizing electron microscope images of internal structures taken under multiple conditions is presented in this paper. Electron microscope images of rubber materials are taken under several conditions, and effective conditions for the prediction of properties are different for each rubber material. Novel approaches for the selection and integration of reliable prediction results are used in the proposed method. The proposed method enables selection of reliable results based on prediction intervals that can be derived by the predictors that are each constructed from electron microscope images taken under each condition. By monitoring the relationship between prediction results and prediction intervals derived from the corresponding predictors, it can be determined whether the target prediction results are reliable. Furthermore, the proposed method integrates the selected reliable results based on Dempster–Shafer (DS) evidence theory, and this integration result is regarded as a final prediction result. The DS evidence theory enables integration of multiple prediction results, even if the results are obtained from different imaging conditions. This means that integration can even be realized if electron microscope images of each material are taken under different conditions and even if these conditions are different for target materials. This nonconventional approach is suitable for our application, i.e., property prediction. Experiments on rubber material data showed that the evaluation index mean absolute percent error (MAPE) was under 10% by the proposed method. The performance of the proposed method outperformed conventional comparative property estimation methods. Consequently, the proposed method can realize accurate prediction of the properties with consideration of the characteristic of electron microscope images described above.

## 1. Introduction

Materials informatics has been attracting attention recently as an important research area for the development of high-performance materials. Modern materials science and engineering research produce a large amount of heterogeneous data, and analyzing the stored data by computational approaches discovers new knowledge for researchers [1,2,3,4]. Among many existing materials, rubber materials’ ability to resist permanent deformation and fracture is well suited for many applications [5]. We can easily list many applications using rubber materials: tires, vibration isolators, seals, hoses, belts, structural bearings, impact bumpers, medical devices, and footwear. These applications involve large static and time-varying strains over long periods. To enhance the performance of the applications, the materials informatics technology for rubber materials is necessary [6,7].

When a rubber material is used for a long time, it usually hardens and degrades and loses its performance. This deterioration is mainly due to heat caused by hysteretic loss, which affects not only the material properties but also the rubber materials’ service life. The performance of such rubber materials can be evaluated by measuring their physical properties through various experiments. Generally, numerical data for various properties of rubber materials such as friction resistance and tensile strength are needed to design and develop rubber materials; however, measurement of the properties is a time-consuming task [8]. If these properties can be determined without measurement procedures, the time required to design and produce high-performance rubber materials can be shortened. Hence, the prediction of rubber material properties has become an important research topic [9].

Methods for prediction of rubber material properties have been intensively studied [10,11,12]. These popular methods use mix proportions or mixing process information for the prediction of properties. It is well known that properties of rubber materials are strongly related to their mixing process information. For example, Liu et al. proposed methods for prediction of Mooney viscosity of rubber materials using mixing process information [11,12]. Mooney viscosity is an evaluation index for the processability, moldability, and flowability of rubber materials, and it can be measured with a specialized device called a viscometer. Mixing temperature, energy, power, pressure, and duration in the chamber of an internal mixer are used as mixing process information for the prediction of Mooney viscosity in those methods. However, some mixing process information such as the order of addition of raw materials in the chamber cannot be quantified.

The mixing process information is reflected in the internal structure of the material and can be visually confirmed. Hence, observation by electron microscope is one of the most important steps in the development phase. Specifically, images of internal structures taken by an electron microscope contain mixing process information regardless of whether the information can be quantified or not. Thus, material property prediction approaches using images of microstructure have been proposed [13,14,15,16]. Machine learning models such as a neural network [17] and relevance vector machines [18] are used in these models, and prediction of material properties is realized with consideration of the internal structures. The advantage of using image information for property estimation is that it allows us to contrast the performance of a material with the structural information of the material that we can intuitively, or visually, understand from electron microscope images.

**Figure 1:**
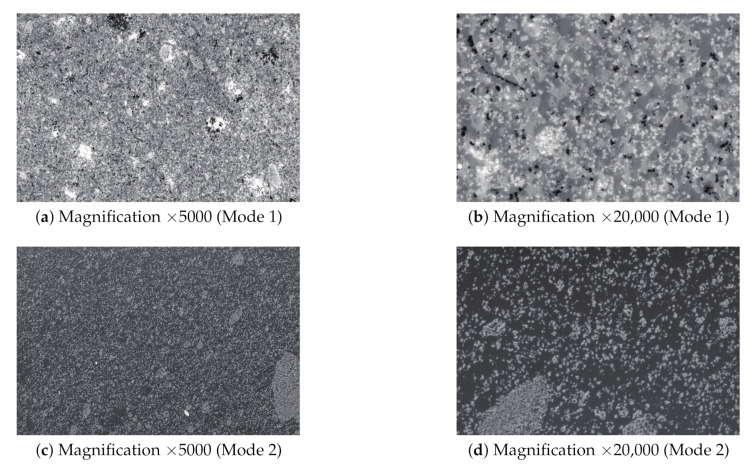
Examples of electron microscope images taken under different conditions: images in (**a**,**b**) were taken with the same imaging condition and images in (**c**,**d**) were also taken with the same imaging condition.

Electron microscope images of rubber materials are generally taken under different conditions such as different magnifications and imaging conditions, as shown in Figure 1. Effective imaging conditions for prediction differ depending on the rubber material. However, only electron microscope images taken under the same conditions have been used in previous methods, and differences in conditions have not been considered. For realization of accurate prediction, it is desirable to construct a predictor with many rubber material samples. Therefore, electron microscope images taken from multiple observations should be used for the prediction of rubber material properties. We should focus on electron microscope images taken under different imaging conditions.

In this paper, we propose a method for prediction of rubber material properties using electron microscope images of internal structures taken under multiple imaging conditions. First, the proposed method selects reliable prediction results from multiple results obtained by predictors that are each constructed from electron microscope images taken under the same condition. This selection process utilizes the prediction interval of the prediction results, which can take into account the accuracy of prediction results [19]. As a result, the proposed method uses only prediction results obtained from electron microscope images taken under beneficial conditions for the subsequent integration. Next, the proposed method integrates these reliable results based on a Dempster–Shafer (DS) evidence theory [20], and this integration result is regarded as a final prediction result. The DS evidence theory has been used to integrate data from multiple information sources, and this theory has been used in many studies as a method for decision level fusion [21,22,23,24]. In the proposed method, information sources of the prediction results are different for each material sample since reliable results are different for each material sample. The DS evidence theory enables successful integration of multiple prediction results, even if the information sources from rubber material samples are different. Consequently, accurate prediction of the properties of rubber materials can be realized by the proposed method.

The contributions of this paper are summarized as follows:

- Our new robust material property prediction method considering different imaging conditions has a wide applicability not only to rubber materials but also to other materials.
- Only reliable prediction results are automatically selected and integrated based on the prediction interval and the DS evidence theory.

This paper is organized as follows. In Section 2, we describe our method for prediction of rubber material properties. In Section 3, experimental results are shown to verify the effectiveness of our method.

## 2. Method for Prediction of Rubber Material Properties

**Figure 2:**
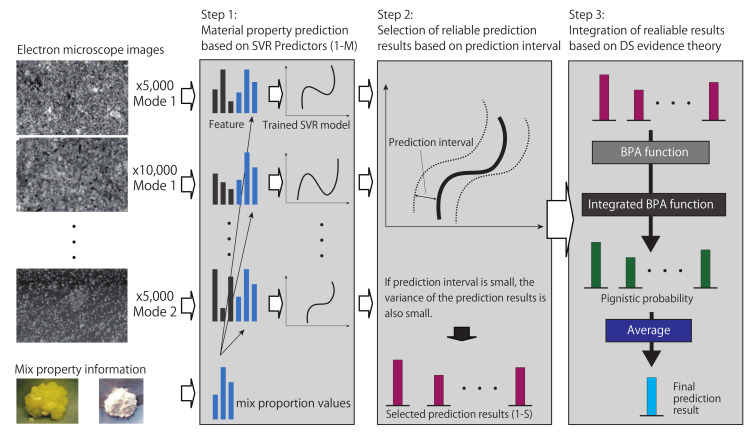
Overview of the proposed method. The proposed method selects *S* (<*M*) reliable prediction results from *M* support-vector-regression (SVR)-based prediction results based on the prediction interval and integrates the reliable results based on the Dempster–Shafer (DS) evidence theory.

In this section, we describe the details of the proposed method. An overview of the proposed method is shown in Figure 2. Our method consists of three Steps (1–3): training of predictors, selection of reliable results, and integration of prediction results. In Step 1, we construct a predictor for each imaging condition of electron microscope images based on the simplest regression model, support vector regression (SVR) [25] using images and mix proportions to obtain multiple prediction results from the predictors. Next, in Step 2, the proposed method selects reliable results from prediction results obtained by these SVR predictors based on prediction intervals [26]. In step 3, basic probability assignment (BPA) functions [27] of these reliable prediction results are determined, and these BPA functions are integrated on the basis of the DS evidence theory to calculate the pignistic probability from these results. Finally, the final prediction result can be simply calculated based on the DS evidence theory.

This section is organized as follows. In Section 2.1, the property prediction method using SVR is explained as Step 1. The process for selection of reliable prediction results in Step 2 is described in Section 2.2. Finally, integration of the selected results based on the DS evidence theory in Step 3 is explained in Section 2.3.

### 2.1. Step 1: Property Prediction for Each Imaging Condition

The proposed method constructs the predictors for each imaging condition based on SVR using the electron microscope images and mix proportions to obtain multiple prediction results. First, the proposed method calculates numerical feature vectors from the electron microscope images and mix proportions. In the electron microscope images, the variation and distribution of luminance change according to the mix proportions and mixing process. Imaging conditions affect the luminance and colors in images. In this study, we have two different image conditions: Mode 1 and Mode 2, as shown in Figure 1. It is common in material development for images to be captured in different conditions and at different magnifications. Therefore, it is necessary to construct a robust method for estimating properties using image information. Therefore, the proposed method utilizes three kinds of numerical features—co-occurrence matrix-related features [28], Gabor wavelet-based features [29] and adaptive local binary patterns [30]—to obtain the visual feature vector by concatenating the above visual features. In the field of general image recognition, although deep-neural-network-based features have been proposed [31], handcrafted features are still useful since the number of electron microscope images is limited and their characteristics are different from those of a pretrained network. The mix proportion feature vectors are each represented as a vector whose elements are mix proportion values. In this way, features for property prediction can be extracted from the set of training images and their corresponding mix proportion information. Since obtained features have high-dimensional vectors, this can easily cause the “curse of dimensionality” when available samples are small. To solve this problem, we utilize the RReliefF algorithm [32] as a feature selection method. The RReliefF algorithm is used to calculate the contribution of each feature to the recognition task, and this also contributes to the dimensionality reduction. We obtain the sophisticated features through the RReliefF algorithm feature selection method. The proposed method obtains a feature vector x by concatenating the visual and mix proportion feature vectors.

Next, the proposed method constructs the predictors based on SVR. In the proposed method, accurate prediction of rubber material properties becomes feasible since SVR enables effective prediction by nonlinear regression even if the number of training samples is small. The SVR calculates the prediction result $\hat{y}$ as follows:

(1) $$\begin{array}{c} \hat{y}=\beta_{p}^{⊤}\Phi \left(x\right)+b_{p}, \end{array}$$

where $\beta_{p}$ is a weighted vector in the property prediction and Φ(·) denotes a nonlinear projection function. In addition, $b_{p}$ is a bias parameter in the property prediction. Nonlinear regression is an extension of linear regression that can be used with large and more general class classification tasks. Unlike linear regression, it can flexibly represent complicated data via a kernel function adopted in the projection function. The SVR determines the optimal weighted vector $\beta_{p}$ by solving the following constrained optimization problem:

(2) $$\begin{array}{c} \begin{array}{cc} \beta_{p}= & \arg\min_{\beta_{p}}\left\{\frac{1}{2}\left|\right|\beta_{p}{\left|\right|}^{2}+C\sum_{i=1}^{N}\left(\xi_{i}+\xi_{i}^{*}\right)\right\},\\ s.t.\; & y_{i}-\beta_{p}^{⊤}\Phi \left(x_{i}\right)-b_{p}\leq \epsilon +\xi_{i},\\ & \beta_{p}^{⊤}\Phi \left(x_{i}\right)+b_{p}-y_{i}\leq \epsilon +\xi_{i}^{*},\\ & \xi_{i},\xi_{i}^{*}\geq 0, \end{array} \end{array}$$

where C(≥0) is a trade-off parameter and $\xi_{i}$ and $\xi_{i}^{*}$ are slack variables. Furthermore, $y_{i}$ is an actual property value of the *i*th (i=1,2,…,N; *N* being the number of training samples) training sample, ϵ is a margin of tolerance, and $x_{i}$ is a feature vector of the *i*th training sample. In this way, we train the *M* SVR predictors for each image condition and magnification that can provide property prediction results. Namely, in the test phase, we can obtain *M* property prediction results in Step 1.

### 2.2. Step 2: Selection of Reliable Prediction Results

We can obtain *M* property prediction results through the processing in Step 1. However, since the SVR is constructed for each imaging condition and magnification, not all the predictions are reliable. In other words, the obtained results include a mixture of reliable and unreliable ones. Therefore, the proposed method adopts the prediction intervals for the selection of reliable prediction results in Step 2. The prediction interval in the regression analysis can take the accuracy of prediction results into account [26]. Specifically, if the prediction interval is small, this means that the variance of the prediction results is also small. Therefore, prediction results for which prediction intervals are smaller than those of other results can be regarded as reliable prediction results.

**Figure 3:**
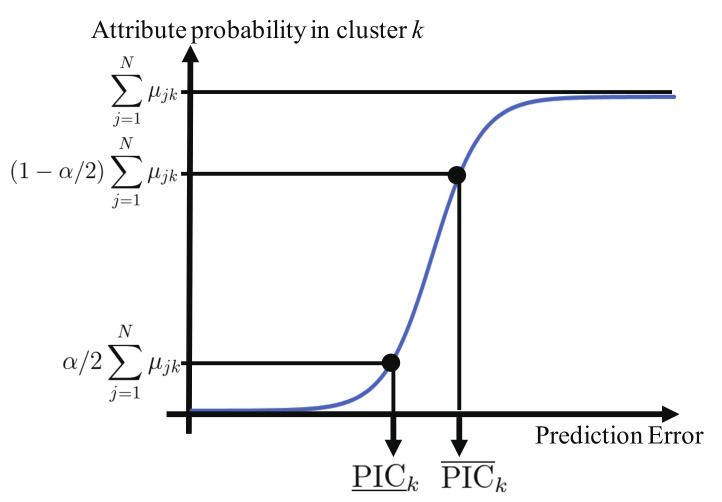
Overview of the empirical distribution function for calculating lower and upper values of the prediction interval in cluster *k*.

First, the proposed method calculates the prediction error $e_{i}$ of the *i*th training sample as follows:

(3) $$\begin{array}{c} e_{i}={\hat{y}}_{i}-y_{i}, \end{array}$$

where ${\hat{y}}_{i}$ is a prediction value of the *i*th training sample. Next, the proposed method applies the fuzzy *c*-means algorithm [33] to the above prediction errors. The proposed method constructs an empirical distribution function, as shown in Figure 3, to calculate lower and upper values of the prediction interval in each cluster.

Specifically, let $\mu_{ik}$ be the *i*th training sample’s attribute probability to the cluster *k* (= 1,2,…,K; K being the number of clusters). ${\tilde{e}}_{i}$ and ${\tilde{\mu }}_{ik}$ are obtained by sorting $e_{i}$ and $\mu_{ik}$ in ascending order, respectively. We denote ${\underline{\mathrm{PIC}}}_{k}$ and ${\bar{\mathrm{PIC}}}_{k}$ as the lower and upper values, respectively, of the prediction interval in cluster *k*. ${\underline{\mathrm{PIC}}}_{k}$ and ${\bar{\mathrm{PIC}}}_{k}$ under the trust rate 100×(1−α)% (0 ≤α≤ 1) are calculated as follows:

(4) $$\begin{array}{c} {\underline{\mathrm{PIC}}}_{k}={\tilde{e}}_{q}, \end{array}$$

(5) $$\begin{array}{c} {\bar{\mathrm{PIC}}}_{k}={\tilde{e}}_{r}, \end{array}$$

where *q* (≤*N*) and *r* (≤*N*) are maximum values to satisfy the following conditions:

(6) $$\begin{array}{c} \sum_{i=1}^{q}{\tilde{\mu }}_{ik}<\alpha /2\sum_{i=1}^{N}\mu_{ik}, \end{array}$$

(7) $$\begin{array}{c} \sum_{i=1}^{r}{\tilde{\mu }}_{ik}<\left(1-\alpha /2\right)\sum_{i=1}^{N}\mu_{ik}. \end{array}$$

The lower and upper values of the *i*th training samples’ prediction result ${\underline{\mathrm{PI}}}_{i}$ and ${\bar{\mathrm{PI}}}_{i}$ are calculated as follows:

(8) $$\begin{array}{c} {\underline{\mathrm{PI}}}_{i}=y_{i}+\sum_{k=1}^{K}\mu_{ik}{\underline{\mathrm{PIC}}}_{k}, \end{array}$$

(9) $$\begin{array}{c} {\bar{\mathrm{PI}}}_{i}=y_{i}+\sum_{k=1}^{K}\mu_{ik}{\bar{\mathrm{PIC}}}_{k}. \end{array}$$

The proposed method calculates the prediction interval of the test samples’ prediction result by utilizing the above prediction intervals of the training samples. Specifically, the prediction interval $\hat{\mathrm{PI}}$ of the test sample is calculated by SVR utilizing the training sample’s prediction intervals ${\mathrm{PI}}_{i}={\bar{\mathrm{PI}}}_{i}-{\underline{\mathrm{PI}}}_{i}$ as follows:

(10) $$\begin{array}{c} \hat{\mathrm{PI}}=\beta_{\mathrm{PI}}^{⊤}\Phi \left(x\right)+b_{\mathrm{PI}}, \end{array}$$

where $\beta_{\mathrm{PI}}$ is an optimal weighted vector calculated from ${\mathrm{PI}}_{i}$ and $b_{\mathrm{PI}}$ is a bias parameter. Then, *S* reliable prediction results for which the prediction intervals are smaller than those of the other results are selected as reliable results from *M* prediction results in our method.

### 2.3. Step 3: Integration of Reliable Results Based on DS Evidence Theory

First, the proposed method determines BPA functions of reliable prediction results that have been obtained as described in the previous section. Then, the proposed method integrates the above BPA functions using the DS evidence theory to calculate the pignistic probability from the integrated result. Finally, the final prediction result can be simply estimated by an average of the selected prediction results for which the values of pignistic probability are higher than a predefined threshold.

Xu et al. proposed a method for determining BPA functions for classification problems [27]. In the proposed method, we extend this determination method to a regression version. First, the property values of the training samples are assigned to *T* clusters using the *k*-medoids algorithm [34]. We define the range in the property domain of cluster *t* (= 1,2,…,T) as $I_{t}$ = [$L_{t}-c\times R_{t}$, $U_{t}+c\times R_{t}$], where $L_{t}$ and $U_{t}$ are, respectively, the lowest value and the highest value of the training samples’ property values belonging to cluster *t*, $R_{t}=U_{t}-L_{t}$, and c∈[0,1]. Next, the probability density function (PDF) of each cluster is calculated. Specifically, the proposed method discretizes the property domain of each cluster into *V* segments. Then, let $p_{tv}$ (v=1,2,…,V) be the probability density of the *v*th discretization domain in cluster *t*, and $p_{t}={\left[p_{t1},p_{t2},\cdots ,p_{tV}\right]}^{⊤}\in R^{V}$. The vector $p_{t}$ is calculated by solving the following constrained optimization problem:

(11) $$\begin{array}{c} p_{t}=\arg\max_{{\tilde{p}}_{t}}\prod_{l=1}^{N_{t}}f\left(y_{tl}|{\tilde{p}}_{t}\right),\\ \;\;\;\;s.t.\;{\tilde{p}}_{tv}\geq 0,v=1,2,\cdots ,V,\\ \;\;\;\;\;\;\;\;\;\;f\left(y|{\tilde{p}}_{t}\right)\geq 0,\forall y\in I_{t},\\ \;\;\;\;\;\;\;\;\;\;\;\;\int f(y|{\tilde{p}}_{t})dy=1, \end{array}$$

where ${\tilde{p}}_{t}={\left[{\tilde{p}}_{t1},{\tilde{p}}_{t2},\cdots ,{\tilde{p}}_{tV}\right]}^{⊤}\in R^{V}$, $y_{tl}$ ($l=1,2,\ldots ,N_{t}$; $N_{t}$ being the number of training samples belonging to cluster *t*) is the property value of the *l*th training sample in cluster *t* and $f\left(y_{tl}|{\tilde{p}}_{t}\right)={\tilde{p}}_{tv}$ if $y_{tl}$ belongs to the *v*th discretization domain in cluster *t*. We solve this constrained optimization problem using a genetic algorithm in the same manner as [35].

We calculate the PDF of each cluster by utilizing the interpolation based on Gaussian process regression (GPR) [36] using the above probability densities $p_{t}$. In the GPR, it is assumed that the available function values represent a particular realization of a Gaussian process. Let $g_{tv}$ be a representative value of segmentation *v* into cluster *t*. The PDF of the *t*th cluster ${\mathrm{Pr}}_{t}\left(y\right)$ is constructed as follows:

(12) $$\begin{array}{c} {\mathrm{Pr}}_{t}\left(y\right)=\left\{\begin{array}{cc} \frac{1}{\sqrt{2\pi \sigma_{t}^{2}}}\exp\left(-\frac{{\left(y-e_{t}\right)}^{2}}{2\sigma_{t}^{2}}\right) & \mathrm{if}\;y\in I_{t}\\ 0 & \mathrm{otherwise} \end{array}\right., \end{array}$$

where $e_{t}$ and $\sigma_{t}^{2}$ are prediction means and variance of cluster *t*, respectively.

The proposed method calculates the test sample’s probability density using the prediction results. Let $\rho_{st}$ be the *s*th (s=1,2,…,S) prediction result’s probability density for cluster *t* obtained by the PDF. We calculate the probability density $\rho_{st}$ as follows:

(13) $$\begin{array}{c} \rho_{st}={\mathrm{Pr}}_{r}\left({\hat{y}}_{s}\right), \end{array}$$

where ${\hat{y}}_{s}$ is the *s*th prediction result of the test sample.

Finally, we determinate the BPA function of each prediction result obtained by SVR as described in the previous section. Let $\theta_{t}$ denote the *t*th identifiable object and let $\Theta \in \{\theta_{1},\theta_{2},\cdots ,\theta_{T}\}$ be a frame of discernment. Let ${\tilde{\rho }}_{s1},{\tilde{\rho }}_{s2},\cdots ,{\tilde{\rho }}_{sT}$ be sorted results in decreasing order of $\rho_{st}$, and $C_{st}\in \Theta$ is the identifiable object that ${\tilde{\rho }}_{st}$ belongs to. If $\exists {\tilde{\rho }}_{st}\neq 0$ (t=1,2,…,T), the BPA function of the *s*th prediction result $m_{s}\left(\cdot \right)$ is determined as follows:

(14) $$\begin{array}{c} \begin{array}{cc} & m_{s}\left(\left\{C_{s1}\right\}\right)={\tilde{\rho }}_{s1},\\ & m_{s}\left(\left\{C_{s1},C_{s2}\right\}\right)={\tilde{\rho }}_{s2},\\ & \;\;\;\;\;\;\;\;\;\;\;\;\;\;\;\vdots \\ & m_{s}\left(\left\{C_{s1},C_{s2},\cdots ,C_{sT}\right\}\right)={\tilde{\rho }}_{sT}. \end{array} \end{array}$$

On the other hand, if $\forall {\tilde{\rho }}_{st}=0$ (t=1,2,…,T), $m_{s}\left(\cdot \right)$ is determined as follows:

(15) $$\begin{array}{c} m_{s}\left(\left\{\theta_{t}\right\}\right)=\exp\left(-b_{t}\right), \end{array}$$

(16) $$\begin{array}{c} b_{t}=\left\{\begin{array}{cc} a_{t}/\sum_{t^{\prime }=1}^{T}a_{t^{\prime }} & \mathrm{if}\max(a_{t})>1\\ a_{t} & \mathrm{otherwise} \end{array}\right., \end{array}$$

(17) $$\begin{array}{c} a_{t}=\frac{1}{S}\sum_{s=1}^{S}\left|{\tilde{y}}_{s}-u_{t}\right|, \end{array}$$

where $u_{t}$ is the representative value of cluster *t*. Then, the proposed method normalizes the obtained BPA function $m_{s}\left(\cdot \right)$ to satisfy the following conditions:

(18) $$\begin{array}{c} \sum_{Q\in \{\left\{C_{s1}\right\},\left\{C_{s1},C_{s2}\right\},\cdots ,\left\{C_{s1},C_{s2},\cdots ,C_{sT}\right\}\}}m_{s}\left(Q\right)=1, \end{array}$$

(19) $$\begin{array}{c} m_{s}\left(\Phi \right)=0, \end{array}$$

where Φ denotes a null set. Therefore, the proposed method normalizes the obtained BPA functions.

Next, the proposed method calculates the final prediction result by integrating the above BPA functions based on the DS evidence theory. The power set of Θ is the set containing all $2^{T}$ possible subsets of Θ represented by P(Θ) defined as

(20) $$\begin{array}{c} P\left(\Theta \right)=\{\Phi ,\left\{\theta_{1}\right\},\cdots ,\left\{\theta_{T}\right\},\left\{\theta_{1},\theta_{2}\right\},\cdots ,\Theta \}. \end{array}$$

According to Dempster’s orthogonal rule [20], we have

(21) $$\begin{array}{c} \begin{array}{cc} m(Y) & \;=\;m_{1}\left(Z_{1}\right)⊕m_{2}\left(Z_{2}\right)⊕\cdots ⊕m_{S}\left(Z_{S}\right)\\ & =\;\frac{\sum_{Z_{1}\cap Z_{2}\cap \cdots \cap Z_{S}=Y}m_{1}\left(Z_{1}\right)m_{2}\left(Z_{2}\right)\cdots m_{S}\left(Z_{S}\right)}{1\;-\;\sum_{Z_{1}\cap Z_{2}\cap \cdots \cap Z_{S}=\Phi }\;\;\;\;\;\;m_{1}\left(Z_{1}\right)m_{2}\left(Z_{2}\right)\cdots m_{S}\left(Z_{S}\right)}, \end{array} \end{array}$$

where $Z_{s}$ is the subset of P(Θ) for the *s*th information source and ⊕ is an operator that represents the integration of BPA functions.

The proposed method calculates the pignistic probability ${\mathrm{Pr}}_{\mathrm{pig}}\left(\theta_{t}\right)$ (t=1,2,…,T) based on the above integrated BPA function m(·) as follows:

(22) $$\begin{array}{c} {\mathrm{Pr}}_{\mathrm{pig}}\left(\theta_{i}\right)=\sum_{D\subseteq \Theta }\frac{|\left\{\theta_{i}\right\}\cap D|\times m\left(D\right)}{\left|D\right|}, \end{array}$$

where |D| is the number of singleton elements in set *D*.

Then, the final prediction result of the proposed method is calculated as the average of reliable prediction results belonging to the cluster that is assigned maximum pignistic probability. Specifically, the proposed method calculates the average value of prediction results belonging to the cluster for which the pignistic probability is higher than those of the other clusters. Let $y_{s^{\prime }}$ be the $s^{\prime }$th (= $1,2,\ldots ,S^{\prime };S^{\prime }$ being the number of prediction results belonging to the cluster having the maximum values of pignistic probability) prediction result obtained by SVR, and $\gamma_{s^{\prime }}\in \Theta$ is the cluster that $y_{s^{\prime }}$ belongs to. The final prediction result ${\hat{y}}_{\mathrm{final}}$ is calculated as follows:

(23) $$\begin{array}{c} {\hat{y}}_{\mathrm{final}}=\frac{1}{S^{\prime }}\sum_{s^{\prime }=1}^{S^{\prime }}y_{s^{\prime }}. \end{array}$$

Since the pignistic probability denotes the confidence of the prediction result, the proposed method enables accurate property prediction.

## 3. Experimental Results

In this section, we show the experimental results to verify the effectiveness of the proposed method. In this experiment, we conducted prediction of rubber material properties using electron microscope images and mix proportions. We used electron microscope images and mix proportions of 75 rubber material samples as the data set. The data set in this experiment consisted of electron microscope images taken under eight different conditions, and the number of samples and electron microscope images in each condition are shown in Table 1. As shown in Table 1, each sample is taken under several conditions, but not all of the conditions are satisfied. Hence, the number of samples and the number of images of each condition are different in the experimental data set. Each electron microscope image was eight-bit grayscale, and its size was 1536 × 1024 pixels.

The proposed method is a novel approach for prediction of properties using electron microscope images of internal structures taken under multiple conditions, and this is the first time that a prediction method using multiple kinds of electron microscope images has been proposed. We compared our method with many conventional methods, including a recently proposed convolutional-neural-network (CNN) [37]-based method. The details of comparative methods used in this experiment are shown in Table 2. According to the proposed method, the kernel function of SVR used in the proposed method and these comparative methods was the Gaussian kernel, and each parameter of the SVR was determined by a practical selection approach in the same manner as the paper [38]. Furthermore, the parameters used in calculation of the prediction interval and the DS evidence theory were determined on the basis of prediction error of the final results. The performance of each method was evaluated via leave-one-out cross-validation. In the leave-one-out cross-validation, a sample was extracted from the 75 samples, the extracted sample was allocated to the test sample, and the other 74 samples were allocated to the training samples. We trained our models using those 74 training samples and estimated the label of the test sample. We repeated this step 75 times to evaluate all samples and calculated the prediction performance.

In order to evaluate the accuracy of the property prediction, we utilized mean absolute error (MAE) and mean absolute percent error (MAPE) shown in the following equations:

(24) $$\begin{array}{c} \mathrm{MAE}=\frac{1}{H}\sum_{h=1}^{H}\left|y_{h}-{\hat{y}}_{h}\right|, \end{array}$$

(25) $$\begin{array}{c} \mathrm{MAPE}=\frac{1}{H}\sum_{h=1}^{H}\frac{|y_{h}-{\hat{y}}_{h}|}{y_{h}}\times 100\;\left(\%\right), \end{array}$$

where *H* (= 75) is the number of test samples.

The experimental results are shown in Table 3. It should be noted that the MAE results provided in Table 3 are the average values of estimated properties for each sample. Namely, the results for 75 test samples are averaged in a single value. From the obtained results, we can confirm that the proposed method realizes the most accurate prediction. Therefore, the effectiveness of the proposed method was verified. A comparison of the results of the proposed method and the results of CM1 shows the effectiveness of using electron microscope images taken under several conditions. We can also confirm that the prediction method using electron microscope images and mix proportions enables accurate prediction of the properties of rubber materials by comparing the results of the proposed method and those of CM2 and CM3. A comparison of the results of the proposed method and those of CM4 and CM5 shows that integration based on the DS evidence theory is effective for prediction of properties of the rubber materials. This is because the pignistic probability denotes the accuracy of each prediction result obtained by the predictor, and successful prediction was realized by integration using these probabilities. Furthermore, we can confirm that the prediction method utilizing selection and integration of reliable results enables accurate prediction of the properties by comparing the results of the proposed method and those of CM6, CM7, and CM8. Finally, we can also confirm that the proposed method enables more accurate prediction than the approaches based on the CNN-based method by comparing the results of the proposed method and those of CM9. This is because the SVR enables accurate prediction even if the number of training sample is small. Considering that the measurement error of the material is always a few percent, it is not too much to say that the proposed method can estimate the properties with high accuracy (less than 10% error).

There are several limitations in this paper. First, although our method can be applied to other materials, we have not confirmed the effectiveness of our method in other materials due to the data restriction. Samples used in this experiment are still small to verify the potential of the proposed method for real-world applications. These concerns should be considered for the next step of our study.

Our rubber material property prediction method is versatile and can be applied not only to rubber materials but also to other materials. In addition, our method is different from the conventional methods in that it can estimate the properties of materials even when the image capturing conditions are not available. In future works, we will improve the accuracy of property estimation by enhancing the image feature extraction methods.

## 4. Conclusions

In this paper, we have proposed a method for prediction of rubber material properties using electron microscope images of internal structures taken under multiple imaging conditions. The proposed method selects reliable prediction results from multiple results obtained by SVR predictors constructed from electron microscope images taken under each condition. The selection utilizes the prediction interval of the prediction results. Then, the proposed method integrates the reliable results based on the DS evidence theory, and this integration result is regarded as the final prediction result. The results of an experiment showed the effectiveness of the proposed method by comparing the results obtained by comparative methods.

## Figures and Tables

**Table 1:** Numbers of samples and electron microscope images in each condition.

Condition	Magnification & Mode	Number of Samples	Number of Images
Condition 1	× 5000, Mode 1	71	71
Condition 2	× 10,000, Mode 1	72	152
Condition 3	× 20,000, Mode 1	72	156
Condition 4	× 40,000, Mode 1	56	118
Condition 5	× 2500, Mode 2	19	19
Condition 6	× 5000, Mode 2	19	38
Condition 7	× 10,000, Mode 2	19	52
Condition 8	× 20,000, Mode 2	19	57

**Table 2:** Comparative methods used in the experiment.

Method	Overview
CM1	The final prediction result is obtained by integration of reliable results using the DS evidence theory. Reliable results are selected from prediction results obtained by an SVR predictor that do not consider different imaging conditions.
CM2	The final prediction result is calculated by integration of reliable results using the DS evidence theory. Reliable results are selected from prediction results obtained by the predictor of each condition using only visual features.
CM3	The final prediction result is obtained by the SVR predictor using only mix proportion features. Each rubber material sample is assumed to have a single mix proportion. Therefore, CM3 does not apply selection and integration of prediction results.
CM4	The final prediction result is calculated by the average of the multiple prediction results.
CM5	The final prediction result is obtained by weighted average of the prediction results. CM5 utilizes prediction interval as the weight.
CM6	The final prediction result is obtained by selection of a reliable result from multiple prediction results using prediction intervals.
CM7	The final prediction result is calculated by the average of reliable results selected from the prediction results obtained by the predictor of each condition using visual and mix proportions features.
CM8	The final prediction result is calculated by integration of multiple prediction results based on the DS evidence theory.
CM9	The final prediction result is calculated by selection and integration of prediction results obtained by a convolutional neural network (CNN) [37]. Specifically, CM9 utilizes Xception [39] that is fine-tuned for property prediction using electron microscope images.

**Table 3:** Mean absolute error (MAE) and mean absolute percent error (MAPE) of prediction results obtained by the proposed method and the comparative methods.

	PM	CM1	CM2	CM3	CM4	CM5	CM6	CM7	CM8	CM9
MAE	**2.68**	4.14	3.77	3.17	2.93	3.04	3.17	2.93	2.69	3.80
MAPE	**9.64%**	15.4%	12.3%	11.7%	11.2%	11.5%	11.7%	11.2%	10.4%	13.7%

## Data Availability

Experimental data cannot be disclosed.
